# Calcific Uremic Arteriolopathy in Hemodialysis Patients: A Report of Two Cases and Review of the Literature

**DOI:** 10.7759/cureus.79885

**Published:** 2025-03-01

**Authors:** Ahmed Amine Jaouahar, Omar Maoujoud, Mohammed Asserraji, Oualid El Filali, Nadir Zemraoui

**Affiliations:** 1 Nephrology and Hemodialysis, Avicenna Military Hospital, Marrakesh, MAR; 2 Vascular Surgery, Avicenna Military Hospital, Marrakesh, MAR

**Keywords:** calcific uremic arteriolopathy, calciphylaxis, cutaneous necrosis, end-stage renal disease, hemodialysis, mineral and bone metabolism disorders, vascular calcifications

## Abstract

Calcific uremic arteriolopathy (CUA), or calciphylaxis, is a rare but life-threatening vascular disorder predominantly affecting patients with end-stage renal disease ongoing hemodialysis. It is characterized by systemic calcification and thrombosis of small arterioles, leading to painful skin necrosis and a high risk of morbidity and mortality. This study presents two cases of hemodialysis patients diagnosed with CUA, highlighting key clinical features, risk factors, and therapeutic challenges. Both patients presented classic predisposing factors, including calcium-phosphorus imbalance, secondary/tertiary hyperparathyroidism, and systemic inflammation. Despite early intervention with phosphate binders, wound care, and dialysis modifications, outcomes varied, reflecting the complexity of disease management. A literature review was conducted to analyze current diagnostic approaches and evolving treatment modalities, emphasizing the need for early diagnosis, multidisciplinary management, and efficient therapeutic strategies. Given the lack of standardized treatment protocols, further research is essential to improve patient outcomes and establish evidence-based guidelines for CUA management in hemodialysis patients.

## Introduction

Calcific uremic arteriolopathy (CUA), formerly known as calciphylaxis, is a rare condition that was diagnosed for the first time in 1961 by Selye et al. [1]. It is a rare but severe complication that affects 4% of patients with end-stage renal disease (ESRD), with a one-year mortality between 45% and 80%, mainly due to septic shock [2]. It involves the obstruction of small cutaneous vessels by phosphocalcic deposits, resulting in extensive and very painful necrotic lesions. Risk factors include obesity, diabetes, and elevated calcium phosphate product. Diagnosis is usually clinical, as skin biopsy is not specific. Histological signs might include media calcification of small and medium-caliber arteries, intimal hyperplasia with fibroblastic and/or giant cell reaction, and thrombosis of subcutaneous vessels. The treatment, not yet clearly standardized, is based on risk factor management such as phosphocalcic balance, improvement of tissue oxygenation, and treatment of pain and skin ulcerations. Specific therapies and molecules such as sodium thiosulfate are increasingly used with promising results.

Our aim is to present two cases of CUA managed in our department, along with a review of recent literature on the topic, to raise awareness about this condition, whose incidence appears to be increasing, especially in the ESRD population.

## Case presentation

Case 1

**Figure 1:**
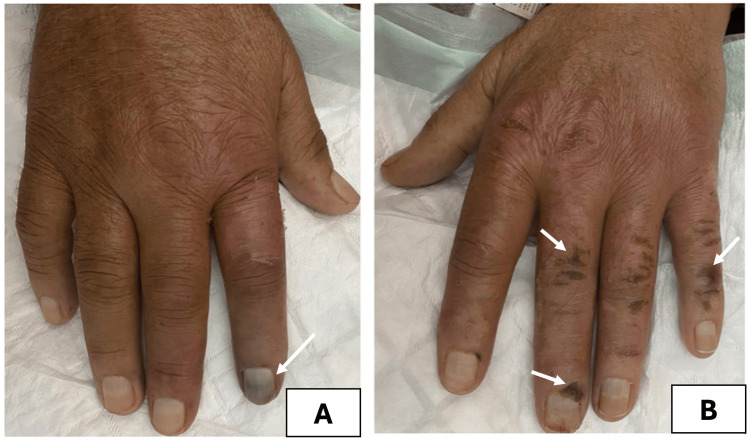
Digital necrosis associated with lesions of calcific uremic arteriolopathy in a chronic hemodialysis patient. A: Circulatory disorders of calcific uremic arteriolopathy mimicking subacute digital ischemia in the right hand. B: Necrosis initially presenting as digital trophic ulcers in the left hand.

The first case was a 71-year-old male with ESRD, who had been on hemodialysis for five years due to severe diabetic nephropathy. The patient had a history of ischemic heart disease and underwent stent placement in the left anterior descending (LAD) artery four years prior, following an acute coronary syndrome. The post-procedural course was uneventful, with follow-up echocardiographic assessments revealing no signs of heart failure. He was admitted to the emergency department and subsequently to the vascular surgery unit for painful, erythematous, ulceronecrotic lesions on the distal hand’s extremities alongside with fever (Figure 1).

Laboratory findings showed normal calcium and phosphorus levels, high alkaline phosphatase levels, and hyperparathyroidism (parathyroid hormone levels more than three times the normal value), along with a slight inflammatory biological syndrome (Table 1).

**Table 1:** Laboratory findings of the patient.

Laboratory parameters	Patient value	Normal range
White blood cells	10.7 x 10^9^	4.0-10 x 10^9^/L
Hemoglobin	10.1	12-16 g/dL
Platelets	380 x 10^9^	150-400 x 10^9^/L
C-reactive protein	55	<0.3 mg/dL
Serum calcium	94	85-105 mg/L
Serum phosphate	43	25-45 mg/L
Alkaline phosphatase	390	24-147 IU/L
Parathormone	202	15-65 pg/mL

**Figure 2:**
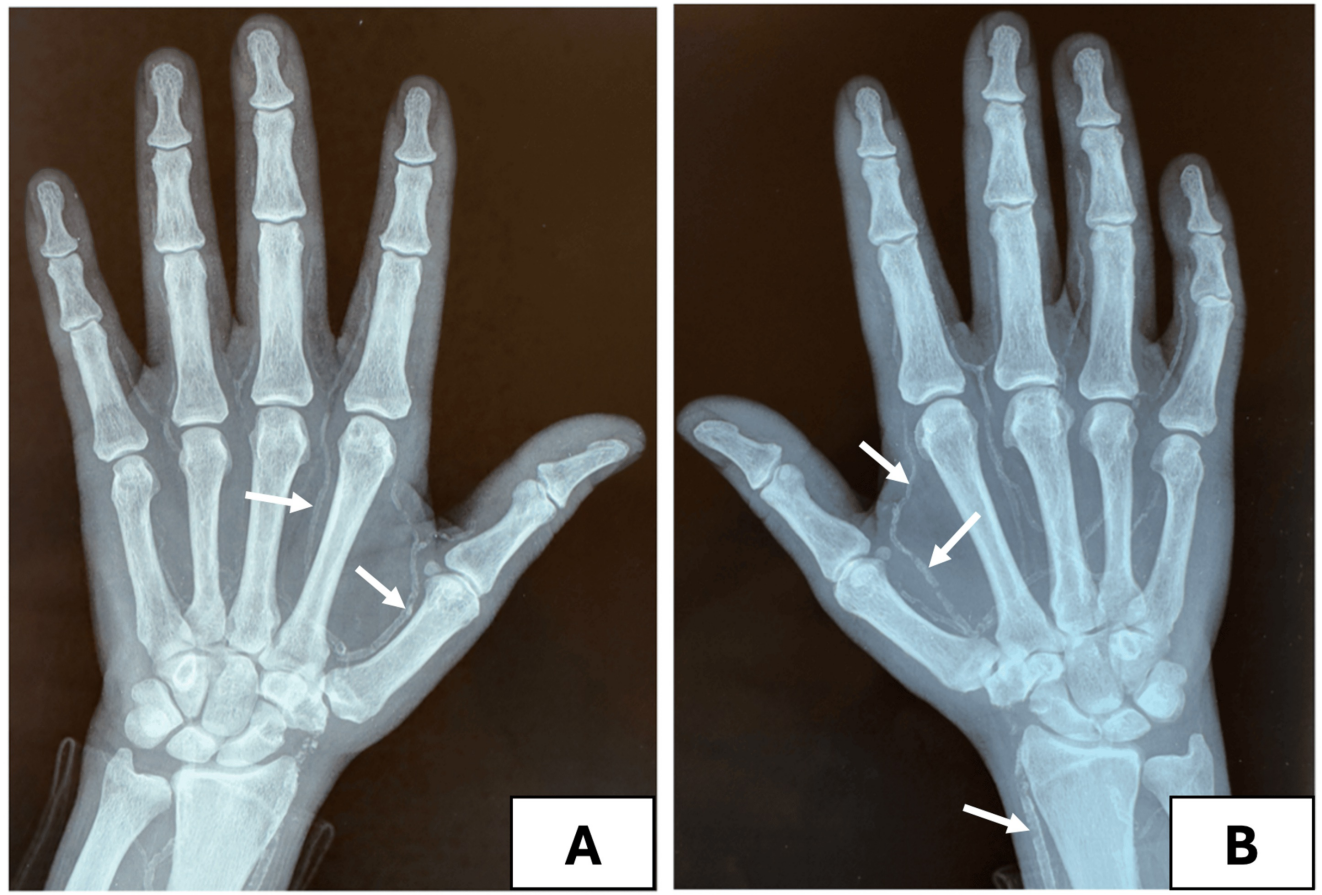
X-ray of both hands showing diffuse arterial calcifications of the upper limb with a net-like pattern observed in the hand vessels. A: Arterial calcifications of the deep palmar arch of the left hand resulting in a net-like pattern. B: Arterial calcifications of the deep palmar arch and the radial artery in the right upper limb.

Radiological examinations revealed a net-like pattern and diffuse medial vascular calcifications on both hands (Figure 2).

**Figure 3:**
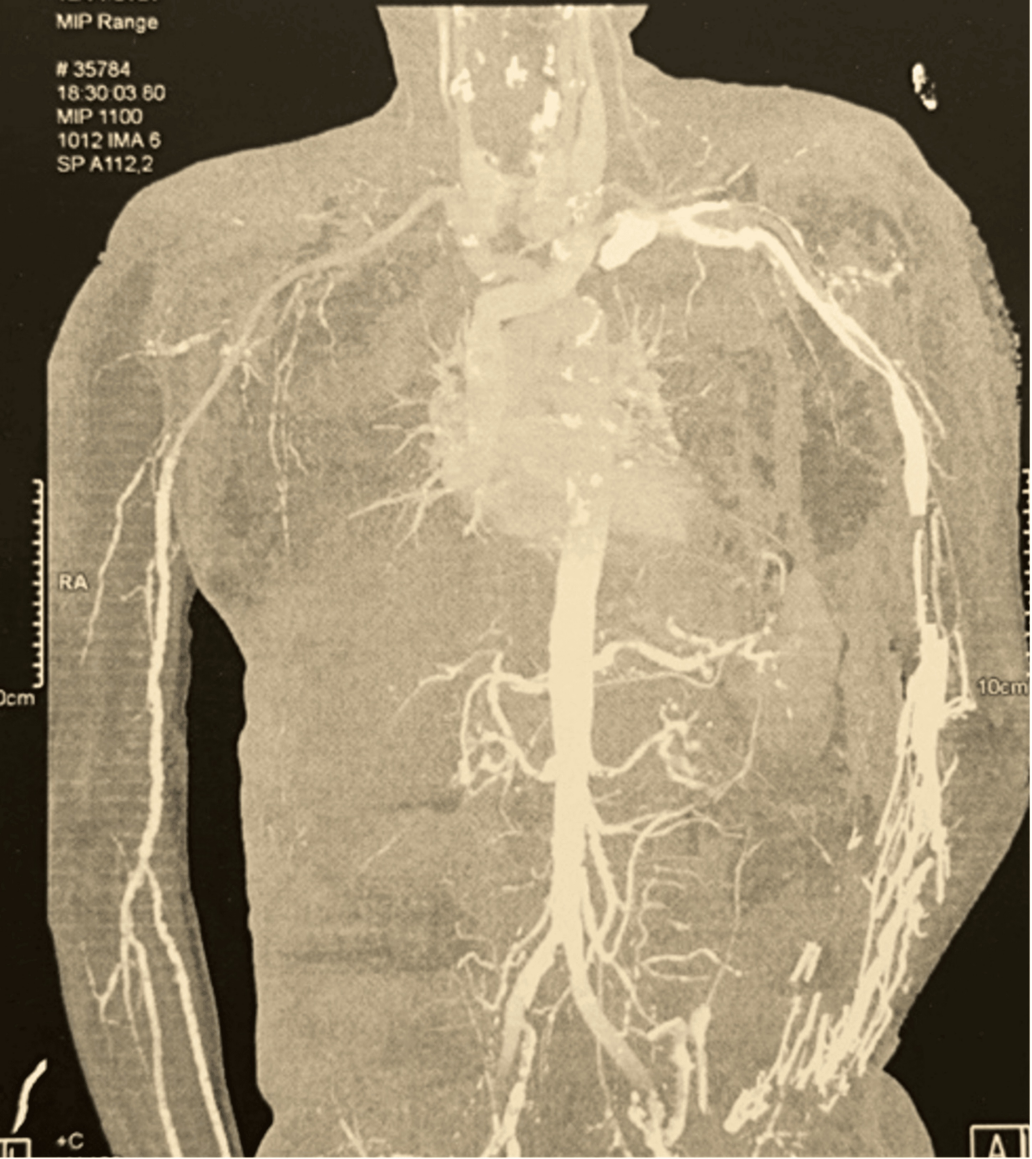
Upper limb CT angiography showing no acute ischemic lesions requiring revascularization.

To assess the possibility of a revascularization procedure, ischemia of the major arterial trunks was ruled out through upper limb CT angiography and conventional angiography (Figure 3).

A skin biopsy confirmed medial calcification of small arteries (hematoxylin and eosin staining) and fat tissue involvement. Based on these findings, a diagnosis of CUA was established. Initial management included debridement and finger amputation, followed by daily hemodialysis, opioid analgesia, and hyperbaric oxygen therapy. The patient showed favorable evolution with progressive regression of the lesions.

Case 2

**Figure 4:**
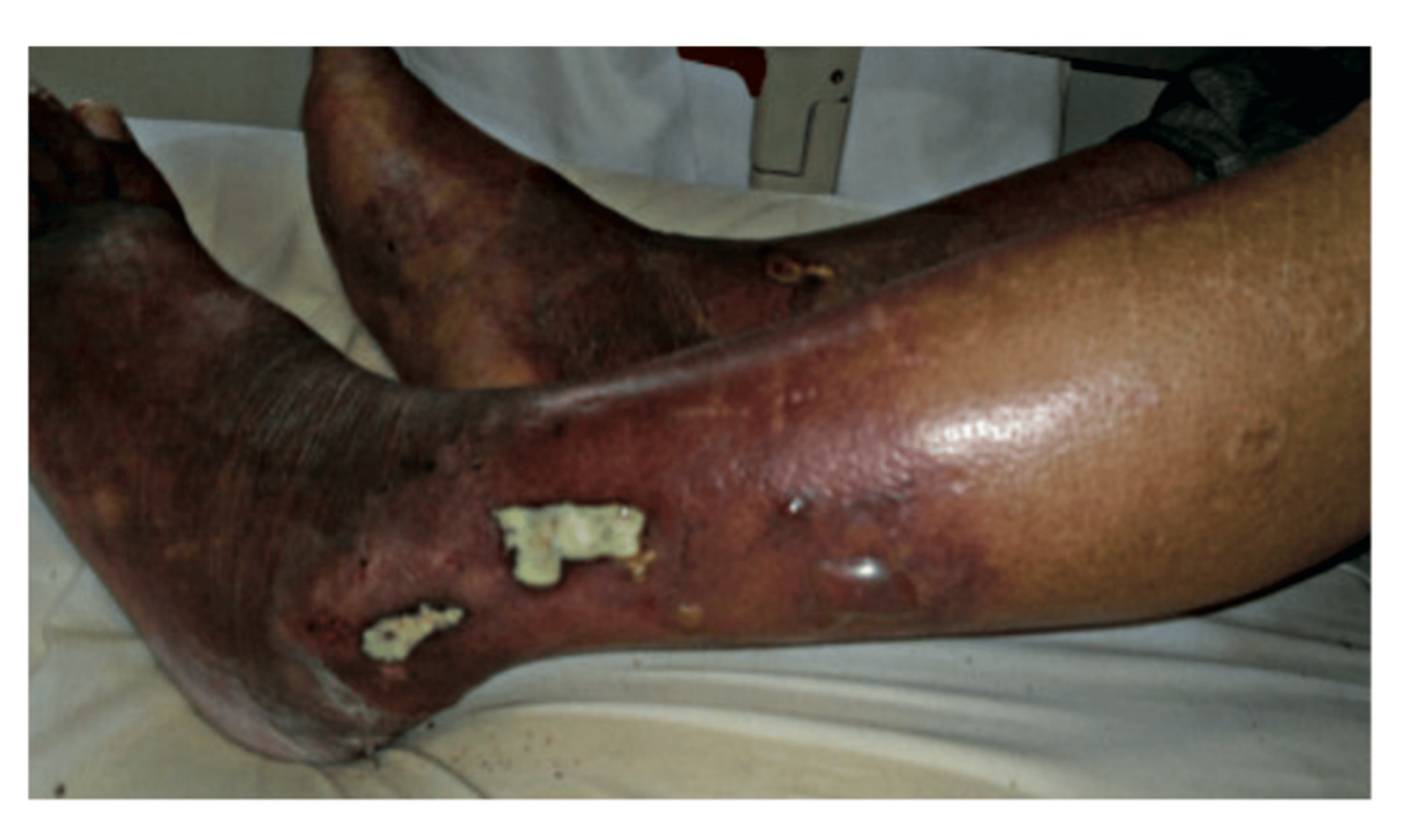
Multiple areas of necrosis and ulceration on the lower limb.

The second case was an 82-year-old patient on hemodialysis for 10 years due to an undetermined nephropathy, with a history of ischemic heart disease and chronic obstructive pulmonary disease (COPD) managed with inhaled corticosteroids (budesonide 200 µg, administered as two inhalations twice daily). He has been admitted to the emergency department and subsequently to the nephrology unit with painful, erythematous, papular lesions on the lower limbs accompanied by fever (Figure 4).

Laboratory findings showed normal calcium and phosphorus levels, a moderate inflammatory biological syndrome, and severe hyperparathyroidism (parathyroid hormone levels more than 10 times the normal value) (Table 2).

**Table 2:** Laboratory findings of the patient.

Laboratory parameters	Patient value	Normal range
White blood cells	9.1 x 10^9^	4.0-10 x 10^9^/L
Hemoglobin	11	12-16 g/dL
Platelets	320 x 10^9^	150-400 x 10^9^/L
C-reactive protein	22	<0.3 mg/dL
Serum calcium	100	85-105 mg/L
Serum phosphate	40	25-45 mg/L
Alkaline phosphatase	310	24-147 IU/L
Parathormone	702	15-65 pg/mL

**Figure 5:**
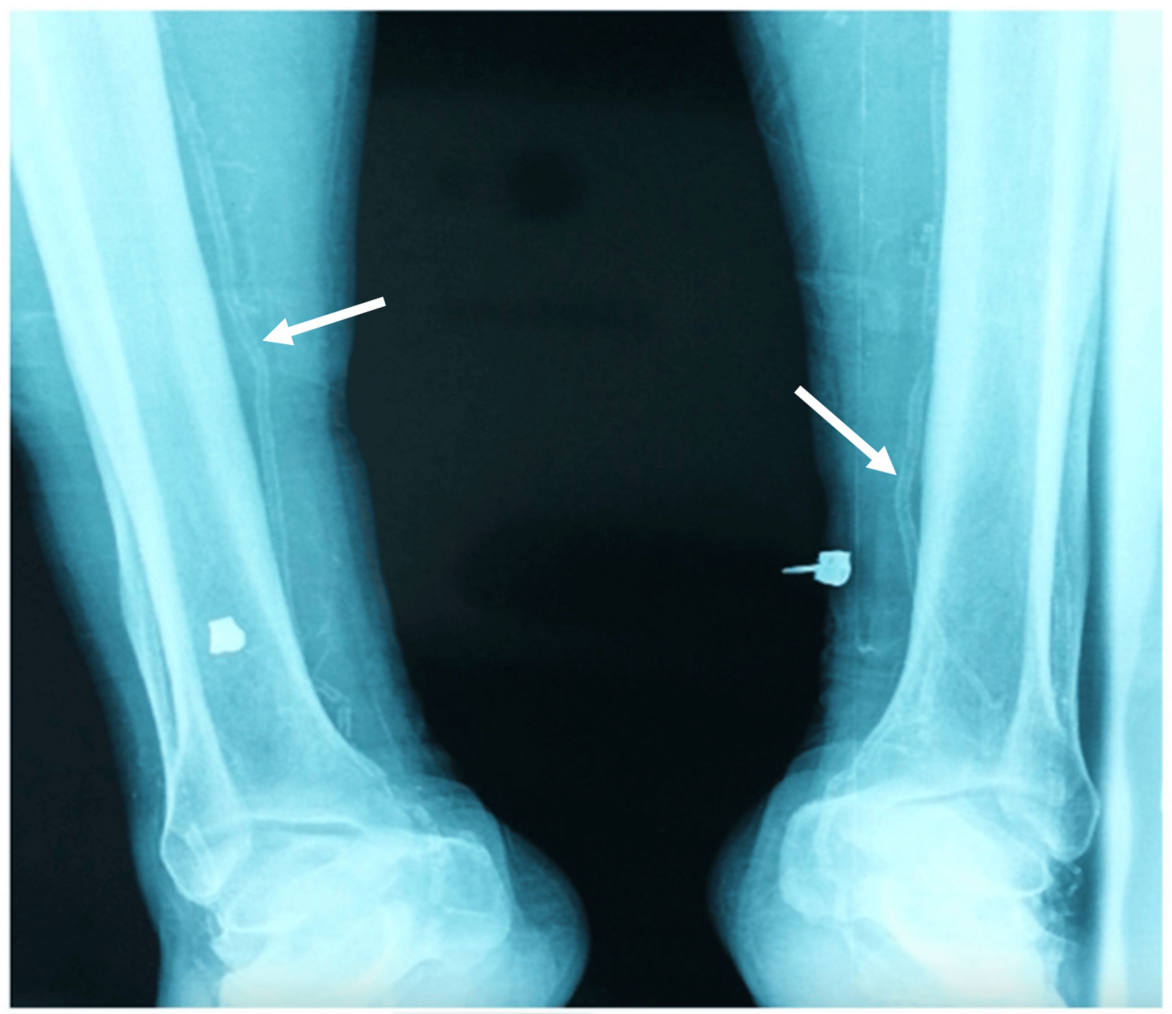
X-ray showing diffuse arterial calcifications of the lower limb arteries.

Radiological examinations revealed diffuse arterial calcifications (Figure 5).

**Figure 6:**
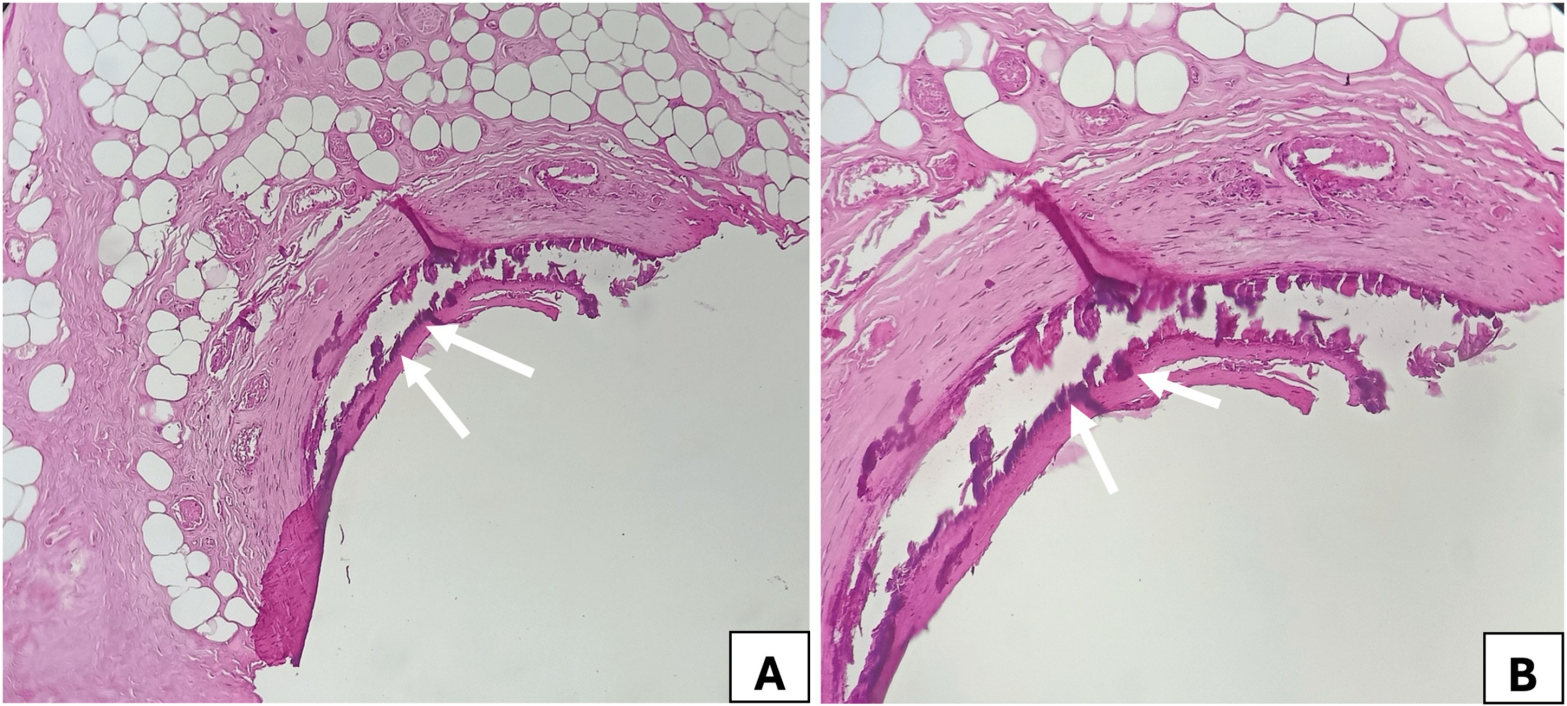
Anatomopathological aspect of a punch skin biopsy of the lower limb: calcium deposits in the medium subcutaneous vessels (indicated by white arrows). A: Hematoxylin and eosin stain, magnification ×100. B: Hematoxylin and eosin stain, magnification ×200.

An initial diagnosis of ecthyma was made, and the patient was treated with antibiotics. However, the lesions progressively extended to both legs. A skin biopsy later confirmed the diagnosis of CUA, revealing calcium deposits in the medial layer of a medium-sized vessel with associated inflammatory cells (Figure 6).

Unfortunately, the patient’s condition deteriorated, and he ultimately succumbed to septicemia caused by *Pseudomonas aeruginosa*, originating from the cutaneous lesions.

## Discussion

CUA is a rare thrombotic microangiopathy that leads to skin necrosis. It is a severe condition with a poor prognosis, most commonly affecting patients with ESRD undergoing hemodialysis or kidney transplant recipients. However, it can also occur in patients without renal failure [3]. Our two patients had ESRD and were ongoing chronic hemodialysis.

The incidence of CUA appears to be increasing for unclear reasons, though it is likely attributed to improved recognition of CUA’s clinical signs and risk factors [2]. CUA is more common in women, with an average age of 48 years across various studies [4]. Studies have suggested the presence of risk factors that promote the development of CUA in ESRD, including hyperphosphatemia, obesity, diabetes, some therapies such as warfarin, and prolonged dialysis duration (greater than 10 years) [5].

The pathogenesis of CUA remains incompletely understood. It is believed to be part of a continuum of systemic vascular and soft tissue calcification, a condition frequently observed in patients with end-stage chronic kidney disease [6]. While large-vessel calcifications are common in patients with ESRD, microvascular skin calcifications leading to clinical manifestations of calciphylaxis remain rare. This rarity may be explained by the predominant involvement of the subcutaneous adipose tissue and the predilection for areas rich in fat. Mature adipocytes exposed to elevated phosphate levels undergo calcification and may also induce vascular smooth muscle cell calcification, likely through the release of adipokines [7].

The suspicion of calciphylaxis is primarily clinical, with cutaneous manifestations being the hallmark, although other signs may also be present, such as painful fat nodules, infiltrated and painful livedo, and ulcerations with a necrotic tendency, especially with bilateral involvement [8]. The condition predominantly affects adipose-rich areas, particularly the abdomen, hips, and buttocks. However, it can also occur in the fingers, toes, breasts, tongue, and penis. Although cutaneous involvement is the most prominent and common, vascular calcifications can also occur in skeletal muscles, the brain, lungs, intestines, eyes, and mesentery.

The definitive diagnosis of calciphylaxis requires a skin biopsy and should be considered whenever this diagnosis is suspected. The biopsy shows a combination of histological lesions, including calcifications, microthromboses, and fibro intimal hyperplasia of small arteries and cutaneous and subcutaneous arterioles. This results in cutaneous ischemia and significant septal panniculitis [9].

Biological tests for the assessment of calciphylaxis typically include total and ionized calcium, phosphorus, alkaline phosphatase (total and bone), parathyroid hormone (PTH), and vitamin D (25-hydroxyvitamin D and 1,25-hydroxyvitamin D). The calcium-phosphate product, which is the product of calcium and phosphorus levels, is often elevated and typically exceeds 70 mg²/dL² in patients with calciphylaxis.

Standard radiography of affected soft tissues reveals subcutaneous vascular calcifications, extravascular calcium deposits, and calcifications of major vascular structures. A reticulated or net-like pattern of calcifications is strongly associated with calciphylaxis, with a specificity of nearly 90%, according to a recent case-control radiological study [10].

The differential diagnosis of CUA includes atherosclerosis, cholesterol crystal embolism disease, vitamin K antagonist (VKA)-induced necrosis, obliterative endarteritis, necrotizing vasculitis, cellulitis, purpura fulminans, oxalate vasculopathy, antiphospholipid syndrome, cardiac myxoma, radiation arteritis, and, in early stages, nephrogenic systemic fibrosis [11,12]. A thorough history, clinical examination, and biological workup can help exclude alternative diagnoses.

Optimal treatment for calciphylaxis remains unstandardized due to the absence of randomized studies and the limited number of reported cases. As a result, treatment recommendations are only based on expert opinion and clinical experience. Management should be multidisciplinary, relying primarily on pathophysiological principles and reported clinical experience in the literature.

The use of sodium thiosulfate (STS) is the cornerstone of the therapeutic strategy. STS functions as a calcium ion chelator via creating calcium thiosulfate, which is subsequently removed in urine or via dialysis. It is believed that STS dissolves calcium deposits in tissues and arteries due to its high solubility. Additionally, it helps neutralize reactive oxygen species (superoxide and hydrogen peroxide ions), thanks to its antioxidant properties. STS also interacts with endothelial nitric oxide (NO) generation to produce a local vasodilatory effect. The administration is intravenous (IV). In hemodialysis patients, the most commonly reported dose is 25 g (two vials of 12.5 g diluted in 100 mL of normal saline), infused over 30 to 60 minutes during the final hour of each dialysis session. For patients weighing less than 60 kg, a reduced dose of 12.5 g is recommended to minimize adverse effects. Local STS application may be considered as an alternative for patients who do not tolerate IV STS. The optimal duration of treatment remains uncertain; however, STS is generally administered for approximately three months. Nevertheless, careful monitoring is required due to the potential risk of fluid and sodium overload in patients receiving STS therapy [13].

Managing underlying or exacerbating risk factors, especially abnormalities in calcium-phosphate metabolism and hyperparathyroidism, represents the following part of treatment. Studies indicate that reducing the calcium-phosphate product has its benefits. Since calcium-based binders may contribute to vascular calcifications, non-calcium phosphate binders, such as sevelamer carbonate, are widely advised for patients with CUA [14].

According to the National Kidney Foundation-Kidney Disease Outcomes Quality Initiative (NKF-KDOQI) standards, it is advised to optimize dialysis prescriptions to achieve adequate dialysis. Preventing peridialytic hypotension and using a low-calcium dialysate (<1.5 mmol/L) are highly recommended. VKAs must be stopped, and heparin treatment may be used as a substitute if necessary. Some studies even suggest vitamin K supplementation [15].

These various treatments can be supplemented with local management of necrotic lesions through surgical debridement, depending on the extent and depth of necrosis. However, routine debridement is not systematically recommended. The role of surgical debridement remains controversial due to the risk of damaging adjacent healthy tissue and the potential for sepsis. It is generally advised for patients with infected wounds, whereas non-surgical management is preferred for non-infected wounds. The primary goal of surgical debridement is to remove necrotic tissue to facilitate wound healing [16].

Patients with severe, progressive CUA or those who fail to achieve wound healing within three months despite the previously mentioned treatments are considered treatment-resistant. In such cases, improving tissue oxygenation based on hyperbaric oxygen therapy (HBOT) is recommended to enhance oxygen delivery to ischemic tissues and promote wound healing [17].

## Conclusions

Calciphylaxis is a rare but severe complication that must be promptly recognized based on its clinical presentation. Despite advancements in therapeutic approaches, treatment responses remain inadequate, and the prognosis of CUA remains extremely poor, with a high mortality rate primarily due to infectious complications, making early diagnosis and prevention crucial in improving patient outcomes.
